# The dual impact: physiological and psychological effects of rapid weight loss in wrestling

**DOI:** 10.3389/fpsyg.2024.1513129

**Published:** 2025-01-06

**Authors:** Barış Sarıakçalı, Fatma Neşe Şahin, Burhan Başoğlu, Levent Ceylan, Özkan Güler, Bade Yamak, Gökhan Arıkan, Gizem Ceylan Acar, Gülşah Sekban, Mehmet Vakıf Durmuşoğlu, Sezen Çimen Polat, Hamza Küçük

**Affiliations:** ^1^Department of Internal Medicine, Faculty of Medicine, Sivas Cumhuriyet University, Sivas, Türkiye; ^2^Department of Coaching Education, Faculty of Sport Sciences, Ankara University, Ankara, Türkiye; ^3^Department of Physical Education and Sport, Faculty of Sport Sciences, Nevşehir Haci Bektaş Veli University, Nevşehir, Türkiye; ^4^Department of Sport Management, Faculty of Sport Sciences, Hitit University, Corum, Türkiye; ^5^Department of Recreation, Yasar Dogu Faculty of Sport Sciences, Ondokuz Mayis University, Samsun, Türkiye; ^6^Mehmet Arabaci School of Physical Education and Sport, Harran University, Sanliurfa, Türkiye; ^7^Department of Sport Management, Faculty of Sport Sciences, Mus Alsarslan University, Mus, Türkiye; ^8^Department of Physical Education and Sport, Faculty of Sport Sciences, Sinop University, Sinop, Türkiye; ^9^Ministry of National Education, Ankara, Türkiye; ^10^Department of Coaching Education, Faculty of Sport Sciences, Gazi University, Ankara, Türkiye; ^11^Department of Physical Education and Sport, Yasar Dogu Faculty of Sport Sciences, Ondokuz Mayis University, Samsun, Türkiye

**Keywords:** wrestlers, rapid weight loss, state-trait anxiety, competition period, psychological

## Abstract

**Introduction:**

Athletes competing in weight-class sports often seek to gain an advantage by competing at lower weights. Athletes competing in weight-class sports often seek to gain an advantage by competing at lower weights. To achieve this, they aim to lose weight during the competition period, leading to various physiological and psychological changes. This study aimed to investigate the biochemical, hormonal, and psychological effects of weight reduction in elite wrestlers during the competition phase.

**Methods:**

Thirty-seven elite male free style wrestlers (age: 19.02 ± 1.27) participated in the study. Samples were collected 5 days before and on the day of the match.

**Results:**

A significant decrease in body weight was observed (*p* < 0.05). Levels of creatine, BUN, sodium, hematocrit, hemoglobin, LDH, and cortisol increased, while albumin, testosterone, and FSH levels decreased. There were no significant differences in potassium, ALT, AST, TSH levels. State and trait anxiety scores of the wrestlers increased significantly during the RWL period.

**Conclusion:**

The study concluded that elite wrestlers experienced significant changes in physiological and psychological parameters during the competition periods. These findings underscore the importance of careful monitoring of RWL strategies by coaches and athletes to mitigate the adverse effects on nutritional status, psychological well-being, and physical performance.

## 1 Introduction

In combat sports, competitions are categorized according to body mass. These categories compete against athletes with the same strength and agility (Artioli et al., 2016). Athletes in the weight category want to compete at lower weights to give themselves an advantage. They want to provide themselves with an advantage with such a strategy (Reale et al., 2017). Athletes who want to compete at a lower weight lose about 2 to 10 percent of their weight, starting 2–3 days before the competition. Athletes use methods that lead to hypohydration or starvation to achieve such a high weight loss (Berkovich et al., 2016; Pettersson and Berg, 2014). RWL is characterized by a 5% weight loss in body weight in less than 1 week. RWL has adverse effects on health-related parameters (Khodaee et al., 2015). Therefore, the impact of weight loss on performance and health should be emphasized. While RWL may offer a competitive advantage to judo athletes in certain cases (Reale et al., 2018), there is often a debate (Lakicevic et al., 2020) about whether its short-term performance benefits are outweighed by the acute and long-term health implications (Kasper et al., 2019). Studies indicate that RWL approaches are not only associated with negative outcomes. However, although some may seem advantageous, the majority of evidence suggests the opposite. The impact of RWL on an individual can be categorized into three subgroups: physical (related to performance), psychological (cognitive), and physiological (indicators).

Strategies for RWL are usually employed to participate in the lightest weight category possible, in order to potentially gain a physical edge over a smaller adversary. Some studies support this idea, as several researches have shown that individuals who shed more body mass experienced greater success in competitions (Pallarés et al., 2016). Athletes who use RWL strategies may experience various physiological effects, including changes in hormonal levels, stunted growth, decreased basal metabolic rate, and immune system dysfunction (Lakicevic et al., 2020). Both short-term and long-term hormonal imbalances have been observed in athletes who engage in RWL. During the competition season, wrestlers consistently show reduced levels of testosterone and insulin-like growth factor 1, according to researchers (Karila et al., 2008; Degoutte et al., 2006). The immune system may be negatively impacted by RWL in athletes, as evidenced by decreased function markers such as T-cells and phagocytic activity (Imai et al., 2002). However, no link has been found between RWL and an increase in injuries or illnesses. A systematic review revealed that RWL may affect kidney function, with combat athletes showing significant increases in blood urea nitrogen and creatinine levels after RWL, potentially indicating acute kidney damage (Coswig et al., 2015; Drid et al., 2019; Kasper et al., 2019; Lakicevic et al., 2021).

There is a growing belief that RWL could harm athletes, and more evidence is indicating the potential drawbacks, particularly in terms of psychological impact. Numerous studies indicate that RWL can negatively impact different aspects of psychological well-being, leading to heightened levels of tension, anger, and fatigue, as well as a significant reduction in vigor (Hiraoka et al., 2019; Isacco et al., 2020). In addition, one major worry linked to athletes using RWL is the risk of developing unhealthy eating habits or eating disorders such as anorexia nervosa and bulimia nervosa.

RWL periods represent critical phases for Olympic style wrestlers, where weight management becomes a key determinant of their athletic performance and competitive outcomes (Roklicer et al., 2022; Lebron et al., 2024). Existing research highlights significant psychological and physiological alterations experienced by wrestlers during these periods. Understanding and anticipating these changes are essential for coaches and athletes to develop effective strategies that optimize physical performance while mitigating potential risks. The objective of this study is to examine the multifaceted physiological and psychological impacts of RWL on wrestlers, contributing to a more comprehensive understanding of this challenging yet crucial aspect of the sport.

## 2 Materials and methods

Thirty-seven male elite free style wrestlers aged 18–20 years participated in the study. The effect size was calculated as 0.60, the significance level as 0.05, and the test power as 0.95 using the G Power program, with a sample size of 32 individuals determined. To avoid statistical errors, the sample group was formed with 37 individuals. The mean age of the participants was 19.02 ± 1.27, and the mean height was 174.64 ± 5.96 cm. Blood parameters were taken 5 days before the championship after 12 h of fasting (first measurement). At the end of 5 days, the last measurement was taken after 12 h of fasting at 08:00, weighting in the morning on the competition date (Table 1).

**Table 1:** Data collection process.

1st Measurement Before Rapid Weight Loss (BRWL)	2nd Measurement After Rapid Weight Loss (ARWL)
5 days before the competition	Competition day (Weight-in morning).
Time: 08:00 (after 12 h of fasting)	
Taking body measurements Collecting blood parameters	

### 2.1 Design and participants

Participants filled out informed consent forms. Afterwards, the responsible investigator collected venous blood samples from the antecubital region in a seated position, and 5.0 mL peripheral blood samples for biochemistry for enzymes were collected in gel tubes. The stability of blood samples was maintained, and cellular and chemical changes were minimized. Blood samples were centrifuged at 3,000 rpm for serum and 2,500 rpm for plasma for 10 min; serum and plasma were separated and analyzed in Sivas Cumhuriyet University Hospital Biochemistry Laboratory.

### 2.2 Instrument and variables

State-Trait Anxiety Index (STAI) Spielberger et al. (1970) measures two dimensions of anxiety via two 20-item instruments. STAI 1 measures state anxiety and transitory feelings of apprehension that are affected by situations. STAI2 measures trait anxiety, the tendency to perceive situations as threatening, and is associated with personality. The STAI state scale is scored on four levels of anxiety intensity from 1, “not at all” to 4, “very much” and with a sum score between 20 and 80, with a higher score indicating higher anxiety (Spielberger et al., 1970; Spielberger and Reheiser, 2009).

### 2.3 Statistical analysis

Before starting the analysis, the assumption of normality was tested. According to the results of the Kolmogorov–Smirnov test (*p* > 0.05), the data followed a normal distribution. A Paired Samples *t*-test was conducted to compare the pre-test and post-test parameters. All statistical calculations were performed using the SPSS 26 software package.

## 3 Results

The findings obtained in the study were presented in Tables 2–5.

**Table 2:** Body weight before and after RWL data.

Parameters	Mean	SD	*t*	*p*	Effect size
Body weight BRWL (kg)	75.43	12.48	27.702	<0.001	0.35
Body weight ARWL (kg)	71.80	11.76			

Wrestlers’ means difference in body weight before and after RWL appeared (*p* < 0.001).

**Table 3:** Changes in biochemical and hematological parameters before and after RWL.

Parameters	Reference range	Mean	SD	*t*	*p*	Effect size
Creatin BRWL mg/dL	0.7–1.2 mg/dL	0.87	0.12	−39.085	<0.001	0.99
Creatin ARWL		1.00	0.14			
BUN BRWL	8–23 mg/dL	13.53	2.52	−32.657	<0.001	0.96
BUN ARWL		16.24	3.02			
Sodium BRWL	136–145 mmol/L	141.54	5.39	−159.632	<0.001	1.27
Sodium ARWL		148.61	5.66			
Hematocrit BRWL	37–47%	47.04	2.92	−97.958	<0.001	1.53
Hematocrit ARWL		51.75	3.21			
Hemoglobin BRWL	12.5–16 g/dL	15.39	2.04	−45.808	<0.001	0.71
Hemoglobin ARWL		16.93	2.24			
Potassium BRWL	3.5–5.1 mmol/L	4.68	0.28	1.678	0.102	0.03
Potassium ARWL		4.69	0.27			
Glucose BRWL	70–100 mg/dL	92.16	5.33	105.163	<0.001	1.81
Glucose ARWL		82.94	4.79			
Albumin BRWL	35–52 g/L	47.02	1.93	18.175	<0.001	2.58
Albumin ARWL		42.24	1.76			
ALT BRWL	0–33 U/L	23.16	9.51	−1.997	0.053	0.01
ALT ARWL		23.27	9.47			
AST BRWL	0–32 U/L	23.70	9.91	−1.882	0.068	0.01
AST ARWL		23.73	9.87			

When the changes in blood biochemical and hematological parameters of wrestlers before and after RWL were examined, a significant difference was found in Creatine, BUN, Sodium, Hematocrit, Hemoglobin, Glucose and Albumin parameters (*p* < 0.01). No significant difference was found in potassium, ALT and AST parameters (*p* > 0.01). Creatine, BUN, sodium, hematocrit, and hemoglobin values increased, while glucose and albumin values decreased after RWL.

**Table 4:** Changes in hormone parameters before and after RWL.

Hormones	Range	Mean	SD	*t*	*p*	Effect size
Testosterone BRWL	2.49–8.36 Mg/L	4.75	1.57	18.376	<0.001	0.48
Testosterone ARWL		4.04	1.33			
LDH BRWL	135–214 U/L	201.27	42.82	−28.589	<0.001	0.65
LDH ARWL		231.46	49.24			
TSH BRWL	0.27–4.2 mIU/L	1.89	0.95	−1.970	0.057	0.04
TSH ARWL		1.93	0.98			
FSH BRWL	1.5–12.4 IU/L	3.82	1.78	8.251	<0.001	0.17
FSH ARWL		3.52	1.68			
Cortisol BRWL	6.02–18.4 ug/dL	11.68	2.65	−26.774	<0.001	0.79
Cortisol_ ARWL		14.01	3.18			

When the changes in hormone parameters of wrestlers before and after RWL were analyzed, a significant difference was found in Total Testosterone, LDH, FSH, and Cortisol parameters (*p* < 0.01). No significant difference was found in the TSH parameter (*p* > 0.05). LDH and cortisol values increased, while total testosterone and FSH values decreased after RWL of wrestlers.

**Table 5:** Comparison of state and trait anxiety in wrestlers.

	*n*	Mean	SD	*t*	*p*	Effect size
Stai 1 Pre	37	38.68	1.72	−10.720	<0.001	1.20
Stai 1 Post		40.62	1.50			
Stai 2 Pre		38.51	1.99	−8.222	<0.001	0.54
Stai 2 Post		39.57	1.92			

State and trait anxiety levels of wrestlers increased during the RWL period.

## 4 Discussion

This study aimed to investigate the physiological and psychological effects of RWL in wrestlers during camp periods. During tournament periods, athletes want to lose weight to compete for the weight they want. If this situation is not applied carefully, some negativities may occur. The study’s main findings were physiological changes in elite wrestlers during periods of RWL. Another finding of our study was that state and trait anxiety levels of wrestlers increased depending on RWL. The body weights of the wrestlers during the RWL period decreased statistically significantly at the end of 5 days. This condition is expected for wrestlers, and it is also a pre-condition for analyzing the RWL effect determined in the study’s hypothesis.

According to the results of the study, significant decreases were found in creatine, BUN, sodium, hematocrit, hemoglobin, glucose, albumin values, which are accepted as indicators of physiological values. Reduced stroke volume due to dehydration is the result of cardiovascular strain (Watanabe et al., 2020). Severe dehydration adversely affects physical and mental performance, and this adverse effect is more significant in hot environments and during long-term exercise (Maughan and Shirreffs, 2010). In this case, BUN, sodium and creatinine values increase, and hemoglobin concentrations increase because blood plasma volume decreases (Walker et al., 1990). This is thought to result from inadequate fluid intake during the RWL of the wrestlers participating in the study. Creatine and BUN levels may increase due to the breakdown of muscle proteins (Riccardi et al., 2013). The energy deficit caused by caloric and fluid restriction during RWL may lead to a greater breakdown of lean body mass, which has a lower energy density compared to body fat, contributing to the overall energy balance (Hall, 2008). In the case of wrestlers, such RWL strategies are often employed to compete in a lower weight class, increasing the likelihood of muscle protein breakdown for energy. Decreased energy intake due to RWL can lead to an energy deficit, lowering blood glucose levels (Yoshino et al., 2020). Albumin is a protein produced in the liver and found in plasma. RWL and decreased protein intake may reduce the liver’s albumin synthesis. When wrestlers’ ALT and AST values are analyzed, there may be no significant change in liver function during RWL (Moman et al., 2022). Lakicevic et al. (2021) emphasized that judokas reduced fluid and food intake and increased exercise frequency during RWL periods. Studies in this period have determined that creatine, creatinine, blood urea nitrogen and urine specific gravity values may vary (Banfi et al., 2009). The biochemical changes in the wrestlers participating in our study may be linked to decreased fluid intake and increased training loads. Performance deteriorates following periods of RWL. There is no universal definition of the changes that occur during periods of RWL, but there is evidence that exercise performance may be reduced (Brechney et al., 2022). Results related to acute dehydration reported a decrease in anaerobic power (Kurylas et al., 2019), anaerobic capacity, maximal strength (Power, and repeated high-intensity effort performance; Alves et al., 2018; Pallarés et al., 2016).

When factors such as the number of competitions, elimination stages, and multiple rounds come into play, it is appropriate to reduce the weight gradually over several weeks. RWL of 3–6% or higher has a negative impact on performance parameters. In addition, perceived fatigue and mood states that occur in the psychophysiology of the athletes cause deterioration in hormonal, blood and urine parameters, body composition and movement kinematics in this process (Martínez-Aranda et al., 2023). Our findings showed that testosterone levels decreased ARWL in wrestlers. Low energy intake and decreased adipose tissue during RWL may adversely affect testosterone production. In addition, high testosterone levels may regulate suppressive effects on cortisol (Rubinow et al., 2005). FSH levels have been found to decrease ARWL in wrestlers. FSH levels may decrease if energy intake is reduced. Sudden energy losses may disrupt the pulsatile release of GnRH (Gonadotropic realizing hormone) from the hypothalamus, decreasing FSH and testosterone levels (Reljic et al., 2015). There was no change in TSH data in wrestlers. TSH may be altered by prolonged energy restriction or weight loss (Agnihothri et al., 2014). However, a significant change in this parameter may have yet to be observed due to the short duration of RWL or individual differences. These results suggest that RWL may cause physiological stress and significantly affect the body’s fluid-electrolyte balance, energy status and protein metabolism. Therefore, athletes’ RWL methods should be carefully managed, and their adverse effects on health should be minimized.

Acute dehydration muscular strength-endurance increases fatigue perception without changes in central or peripheral function markers. The perception of fatigue during this process causes a decrease in muscle performance (Barley et al., 2018; Stewart et al., 2014). Fatigue-related processes often accompany significant hormonal changes, negatively affecting physical performance (Küçük and Ceylan, 2022). These alterations in hormone levels may hinder an individual’s ability to sustain physical activity, leading to reduced endurance and strength. Similar effects can be observed in psychological processes, where hormonal imbalances (Kucuk et al., 2024) may contribute to cognitive decline, mood disturbances, and a decrease in overall mental well-being (Ali et al., 2018).

RWL has psychological effects as well as a decrease in the performance of athletes. Depression and anger develop in athletes due to weight loss in this process (Marttinen et al., 2011). Tension and fatigue are seen before the competition (Degoutte et al., 2006). Furthermore, during the RWL period, athletes may feel in different emotional states. It was found that 60% of university student wrestlers were angry during the RWL period (Sundgot-Borgen and Garthe, 2011). The wrestlers who participated in our study were found to have trait and state anxiety during the RWL period. This finding shows that athletes feel anxiety about being successful in matches. The athlete struggling to lose weight must also show superior performance. In this period, athletes should be meticulous and prepared for training, nutrition and dehydration.

Some studies suggest a negative relationship between changes in testosterone levels and anxiety. One study indicated that testosterone replacement therapy can reduce anxiety (Cooper and Ritchie, 2000). Another study demonstrated that elevated testosterone levels in male rats were associated with decreased anxiety. Conversely, a different perspective posits that there is no relationship between testosterone levels and anxiety (Frye and Seliga, 2001; Aikey et al., 2002). Contrary to these claims, a study conducted with university students identified a relationship between low testosterone levels and increased anxiety levels (Granger et al., 2003). Our results indicate that testosterone levels decrease during the RWL period while anxiety levels increase. This increase in anxiety may not be related to hormonal changes but could be associated with the anxiety state that arises during the RWL phase.

RWL is an essential period for athletes. Problems that occur during this period will increase metabolic risks and negatively affect athletes’ careers (Drid et al., 2024). Studies indicate that weight loss should not exceed 5% (Lakicevic et al., 2024; Janiszewska and Przybyłowicz, 2020). The literature characterizes RWL as a reduction of 5% or more of body weight within a period of less than 1 week, noting that such practices can lead to considerable negative effects on athletes’ physical, physiological, and psychological well-being (Khodaee et al., 2015). Although it is not an exact expression as a ratio, this process should be managed well. To protect against the negative effects of the RWL period, athletes can seek psychological support and receive nutrition education to apply proper weight loss strategies. Coaches should also emphasize the importance of balanced training loads and adequate recovery, ensuring that the athletes’ well-being is prioritized throughout the RWL phase. Integrating these measures will support both the mental and physical health of athletes during intense training and weight loss periods.

As a result, it was determined that elite wrestlers had changes in biochemical, hormone and psychological values during RWL periods.

## 5 Limitations and strengths

The limitation of this study is that it focuses on the wrestling branch. Another limitation is that similar conditions were followed after the RWL period. Future studies should be conducted in different branches, covering different time intervals.

## Data Availability

The raw data supporting the conclusions of this article will be made available by the authors, without undue reservation.
