# Erratum: The dual impact: physiological and psychological effects of rapid weight loss in wrestling

**DOI:** 10.3389/fpsyg.2025.1568675

**Published:** 2025-02-11

**Authors:** Frontiers Production Office

**Affiliations:** Frontiers Media SA, Lausanne, Switzerland

**Keywords:** wrestlers, rapid weight loss, state-trait anxiety, competition period, psychological

Due to a production error, the affiliations for the authors Burhan Başoğlu and Sezen Çimen Polat were incorrect. Instead of ‘Department of Physical Education and Sport, Faculty of Sport Sciences, Sinop University, Sinop, Türkiye', Burhan Başoğlu should be affiliated to ‘Department of Physical Education and Sport, Faculty of Sport Sciences, Nevşehir Haci Bektaş Veli University, Nevşehir, Turkiye'. Similarly, instead of ‘Department of Coaching Education, Faculty of Sport Sciences, Ankara University, Ankara, Türkiye', Sezen Çimen Polat should be affiliated to ‘Department of Coaching Education, Faculty of Sport Sciences, Gazi University, Ankara, Türkiye'. The numbering of the remaining affiliations has been adjusted accordingly.

The complete updated affiliation details are shown below.

Bariş Sarıakçalı^1^, Fatma Neşe Şahin^2*^, Burhan Başoğlu^3^, Levent Ceylan^4^, Özkan Güler^2^, Bade Yamak^5^, Gökhan Arıkan^6^, Gizem Ceylan Acar^7^, Gülşah Sekban^8^, Mehmet Vakıf Durmuşoğlu^9^, Sezen Çimen Polat^10^ and Hamza Küçük^11^

^1^ Department of Internal Medicine, Faculty of Medicine, Sivas Cumhuriyet University, Sivas, Türkiye

^2^ Department of Coaching Education, Faculty of Sport Sciences, Ankara University, Ankara, Türkiye

^3^ Department of Physical Education and Sport, Faculty of Sport Sciences, Nevşehir Haci Bektaş Veli University, Nevşehir, Turkiye

^4^ Department of Sport Management, Faculty of Sport Sciences, Hitit University, Corum, Türkiye

^5^ Department of Recreation, Yasar Dogu Faculty of Sport Sciences, Ondokuz Mayis University, Samsun, Türkiye

^6^ Mehmet Arabaci School of Physical Education and Sport, Harran University, Sanliurfa, Türkiye

^7^ Department of Sport Management, Faculty of Sport Sciences, Mus Alsarslan University, Mus, Türkiye

^8^ Department of Physical Education and Sport, Faculty of Sport Sciences, Sinop University, Sinop, Türkiye

^9^ Ministry of National Education, Ankara, Türkiye

^10^ Department of Coaching Education, Faculty of Sport Sciences, Gazi University, Ankara, Türkiye

^11^ Department of Physical Education and Sport, Yasar Dogu Faculty of Sport Sciences, Ondokuz Mayis University, Samsun, Türkiye

The publisher apologizes for this mistake.

The original version of this article has been updated.

